# Surgical-site infection indices detected by post-discharge surveillance in a medium sized hospital in the city of São Paulo, Brazil

**DOI:** 10.1186/1753-6561-5-S6-P193

**Published:** 2011-06-29

**Authors:** DP Cais, F Minenelli, K Tonelli, P Rebelo

**Affiliations:** 1 Infection Control, Hospital Samaritano, São Paulo, Brazil

## Introduction / objectives

Surgical-site infections (SSI) account for about 24% of hospital infections. Due to short postoperative staying, SSI diagnosis is eventually made after discharge. SSI rates may be under reported; therefore surveillance after discharge is needed to obtain reliable indices of SSI and to improve quality of care. **Aim:** To describe the SSI rate assessed after discharge and to compare post-discharge rates to intra hospital rates.

## Methods

This is a retrospective analysis of data collected between September 2009 and December 2010 in a medium sized private hospital in the city of Sao Paulo. Active surveillance after discharge is a governmental requirement and was performed by telephone. We used a standard questionnaire to investigate the occurrence of signs and symptoms of infection: pain, swelling, redness, warmth, fever, presence of secretion and nodules around the incision. Once the SSI was identified, its occurrence was notified and the patient was followed by 60 and 90 days, by telephone.

## Results

From 5,414 surgical patients, 5,213 (96.3%) agreed to answer the questionnaire. SSI rate was 2.4% (129/5,414): there were 88 (68.2%) intra hospital SSI and 41 (31.8%) cases identified post-discharge. No suspected cases of *Mycobacterium spp*. infection were identified.

## Conclusion

The post-discharge infection rate highlights the importance of a follow up. For institutions that do not have outpatient clinics, post-discharge surveillance is required. Amongst other methods, telephone contact seems to be a reliable strategy since it is possible to assess a large number of patients, although costs and feasibility need to be considered before its implementation.

## Disclosure of interest

None declared.

